# Global research trends in robotic applications in cardiovascular medicine: a bibliometric analysis

**DOI:** 10.1007/s11701-026-03629-6

**Published:** 2026-07-20

**Authors:** Ozan Oğuz, Mehmet Akif Erdöl, Çağrı Yayla, Ahmet Göktuğ Ertem

**Affiliations:** 1 Department of Cardiology, Sakarya Yenikent State Hospital, Sakarya, Turkey; 2 Department of Cardiology, Ankara Bilkent City Hospital, Ankara, Turkey

**Keywords:** Robotic surgery, Cardiovascular medicine, Bibliometric analysis, Coronary artery bypass grafting, Percutaneous coronary intervention

## Abstract

This study aimed to analyze global research trends in robotic applications in cardiovascular medicine. Robotic technologies have been increasingly integrated into cardiovascular medicine, providing enhanced precision, improved visualization, and reduced operator-related risks. Despite growing interest in this field, comprehensive evaluations of global research trends remain limited. In this bibliometric study, publications indexed in the Web of Science Core Collection between 2019 and 2025 were systematically analyzed, and 261 original articles meeting predefined criteria were included. Bibliometric analyses were carried out using VOSviewer and Biblioshiny to evaluate publication trends, citation dynamics, leading sources, and collaboration patterns. Scientific output showed a steady upward trajectory over time, with a more pronounced increase after 2021 and reaching its peak in 2025. The highest average citation rate was observed in 2024. *Journal of Robotic Surgery* and *Journal of Cardiac Surgery* were identified as the most productive journals. The United States was the leading contributor in terms of both publication volume and citation impact and also played a central role in international collaborations. Keyword analysis showed that research activity was primarily focused on *robotic surgery*, *minimally invasive surgery*, *coronary artery bypass grafting*, and *percutaneous coronary intervention*. Overall, robotic applications in cardiovascular medicine represent a rapidly expanding research area characterized by increasing scientific output and growing international collaboration. The concentration of publications in specific journals and the prominence of leading countries suggest a developing and increasingly structured research landscape.

**Supplementary Information:**

The online version contains supplementary material available at 10.1007/s11701-026-03629-6.

## Introduction

Globally, cardiovascular diseases constitute a major source of both morbidity and mortality. Advances in diagnostic and therapeutic technologies, particularly the development of minimally invasive approaches, have significantly transformed clinical practice. Among these advancements, robotic systems have emerged as a key component of this transformation, offering enhanced precision, improved visualization, and superior maneuverability in both surgical and interventional procedures, thereby gaining an increasingly important role in cardiovascular medicine [1].

Robotic applications have demonstrated promising outcomes in areas such as robotic coronary artery bypass grafting (CABG), valvular surgery, and robot-assisted percutaneous coronary interventions (RA-PCI). In particular, RA-PCI has been shown to reduce operator radiation exposure, fluoroscopy time, and contrast usage, while maintaining clinical outcomes comparable to those of manual PCI [2]. Although robotic surgery has also been associated with favorable short-term outcomes [3], its widespread adoption remains limited due to factors such as high cost, a significant learning curve, the need for experienced multidisciplinary teams, and certain technical constraints. Notably, effective implementation of robotic surgery requires appropriate patient selection, standardized procedural techniques, and structured training programs [4]. Nevertheless, these limitations are gradually becoming more manageable with ongoing technological advancements and increased clinical experience.

Although robotics-related bibliometric studies have been published, comprehensive analyses specifically focused on cardiovascular medicine remain limited. A systematic assessment of the current literature can offer important insights into the present landscape of robotic applications in cardiovascular medicine and guide future research in this rapidly advancing field. Accordingly, this study sought to conduct a comprehensive bibliometric analysis of global research trends in robotic cardiovascular applications, with a particular focus on publication patterns, key research themes, leading countries, and scientific collaboration networks.

## Materials and methods

Data for this bibliometric study were obtained from the Web of Science (WoS) Core Collection database. The search terms were developed based on a review of the existing literature, commonly used terminology in robotic cardiovascular medicine, and iterative pilot searches performed within the WoS database. A systematic search of the literature was carried out through the “topic” (TS) field, based on the following search strategy: TS = ((“robotic” OR “robot-assisted” OR “robotic-assisted” OR “robotic surgery” OR “robotic intervention” OR “robotic system” OR “robotic platform” OR “medical robotics” OR “surgical robotics” OR “robot-assisted surgery” OR “robotic catheterization” OR “robotic navigation” OR “robotic angiography” OR “robotic percutaneous coronary intervention” OR “robotic PCI” OR “robot-assisted percutaneous coronary intervention” OR “robot-assisted PCI” OR “robotic coronary intervention” OR “robotic angioplasty” OR “CorPath” OR “CorPath GRX”) AND (“cardiovascular” OR “cardiac” OR “heart” OR “coronary” OR “cardiology” OR “cardiovascular medicine” OR “cardiovascular surgery” OR “cardiac surgery” OR “cardiothoracic surgery” OR “coronary artery disease” OR “CAD” OR “coronary revascularization” OR “myocardial revascularization” OR “coronary artery bypass grafting” OR “CABG” OR “coronary artery bypass” OR “percutaneous coronary intervention” OR “PCI” OR “angioplasty” OR “coronary angioplasty” OR “coronary angiography” OR “structural heart disease” OR “valve surgery” OR “valve repair” OR “heart valve surgery” OR “transcatheter aortic valve implantation” OR “TAVI” OR “transcatheter aortic valve replacement” OR “TAVR” OR “transcatheter valve intervention”)). The timespan was defined as 2019–2025, and all data were retrieved on April 26, 2026. The study period was restricted to 2019–2025 to focus on contemporary research trends and recent developments in robotic cardiovascular medicine during a period of rapidly increasing scientific activity.

**Fig. 1:**
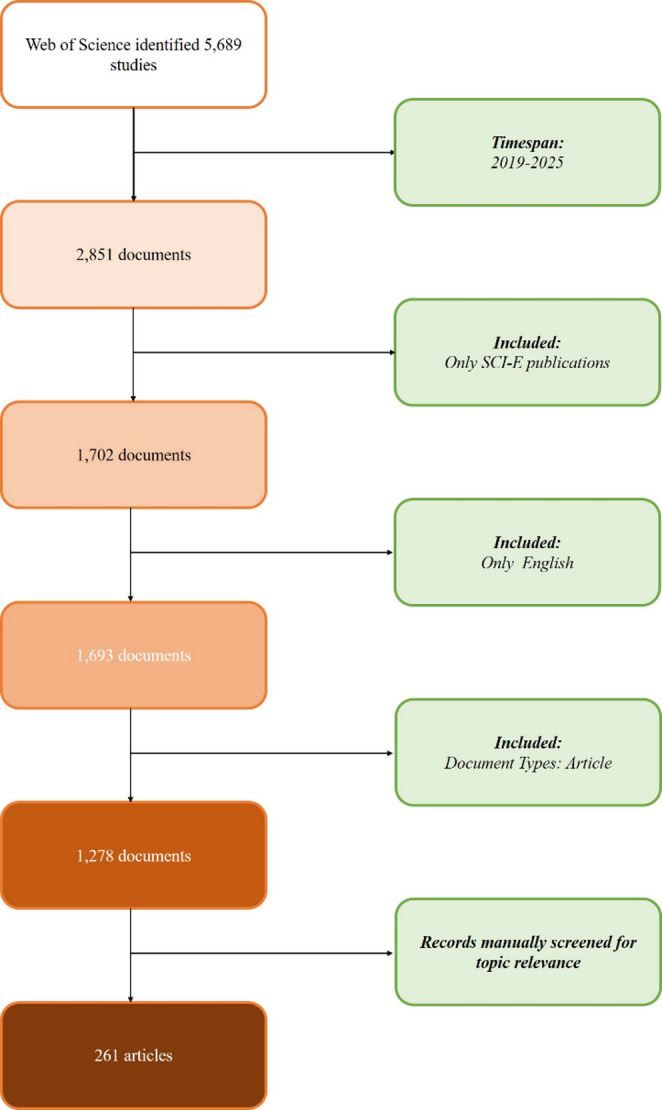
Flowchart illustrating the study selection process (SCI-E: Science Citation Index Expanded)

The initial search identified 5,689 records in the Web of Science database. After applying the predefined timespan filter (2019–2025), 2,851 publications remained. Subsequently, the dataset was limited to studies indexed in the Science Citation Index Expanded (SCI-E), yielding 1,702 records. Further restriction to English-language publications resulted in 1,693 studies. Finally, only documents classified as “articles” were included, leaving a total of 1,278 studies for analysis (Fig. 1). Only original articles were included to ensure methodological consistency and to focus on primary research contributions. Reviews, editorials, conference papers, guidelines, and consensus statements were excluded because these publication types may have substantially different citation behaviors and could disproportionately influence bibliometric indicators.

After retrieval, each publication was screened for relevance according to predefined eligibility criteria. Studies were included if they were original research articles directly related to robotic applications in cardiovascular medicine, including robotic cardiac surgery, robot-assisted coronary interventions, and related cardiovascular robotic technologies. Publications focusing on robotic technologies without direct cardiovascular clinical relevance were excluded. Examples of excluded categories included cerebrovascular interventions, non-cardiovascular surgical applications, rehabilitation technologies, radiotherapy-related applications, and other unrelated robotic fields. A total of 261 original research articles meeting the study criteria were ultimately included in the bibliometric analysis. The screening procedure was carried out independently by two authors (O.O. and M.A.E.), with discrepancies resolved through discussion until agreement was achieved; when needed, a third author (Ç.Y.) provided the final judgment.

All bibliometric analyses were conducted using Biblioshiny (version 4.1.3) and VOSviewer (version 1.6.20), while Microsoft Excel 2016 was used for data management and preliminary organization [5–8]. The study design, analysis, and reporting were informed by commonly accepted methodological approaches used in bibliometric research. Biblioshiny was used to generate annual scientific production, average citations per year, source analyses, source production over time, reference publication year spectroscopy (RPYS), most globally cited documents, affiliation analyses, corresponding authors’ country analyses, country citation analyses, and the international country collaboration map. VOSviewer was used to construct and visualize country co-authorship, author co-authorship, and keyword co-occurrence networks. The combined use of both software packages enabled complementary analytical and visualization approaches, providing a more comprehensive evaluation of the research landscape.

Research productivity over time was evaluated based on annual publication counts. Citation impact was assessed using average citations per year to account for differences in publication time and to provide a normalized comparison across years.

The analysis encompassed the assessment of key sources, their temporal production patterns, RPYS, the most globally cited documents, and leading affiliations. Trends in source productivity and their evolution over time were further examined to identify the most influential journals in the field. RPYS was conducted to explore the historical development and foundational studies in the field. In RPYS, citation peaks were used to identify influential references that contributed to the historical development and intellectual foundation of the field. Additionally, the most globally cited documents were analyzed to determine the most influential publications, while institutional contributions were evaluated through analysis of the most relevant affiliations.

Country-level bibliometric analyses were conducted to assess global research output and collaboration patterns. Corresponding author countries were classified as single-country publications (SCP) and multiple-country publications (MCP), representing domestic and international collaborations, respectively. A country collaboration map was constructed by including only connections supported by at least four co-authored publications to improve clarity. The most highly cited countries were determined based on total citation counts. In addition, a country-level co-authorship network was constructed using VOSviewer, in which node size represented the contribution of each country and links reflected the strength of collaboration, with inclusion criteria defined as a minimum of one document and zero citations.

VOSviewer was employed for network mapping. Patterns of co-authorship and keyword co-occurrence were evaluated using a full counting scheme combined with association strength–based normalization. In the generated maps, entities were displayed as nodes and their connections as links. The extent of network connectivity was quantified through total link strength (TLS).

Author-level co-authorship patterns were analyzed using both network and overlay visualization techniques. In these representations, node size corresponded to each author’s relative contribution, while the connections between nodes reflected collaboration intensity. Average publication year (APY) was used to construct the overlay visualization. Inclusion criteria were defined as a minimum of two documents and zero citations.

The conceptual framework of the field was examined using keyword co-occurrence analysis derived from author-defined keywords. In the network maps, keywords were depicted as nodes, while the connections between them represented their co-occurrence within the same publications. The size of each node corresponded to the frequency of keyword usage, and a minimum threshold of two occurrences was applied for inclusion. Additionally, a treemap visualization was created for keywords appearing at least five times.

Before conducting the analyses, the dataset underwent an extensive cleaning and preprocessing stage. To ensure uniformity in VOSviewer outputs, separate thesaurus files were created and applied for author names (Supplementary Material 1), country names (Supplementary Material 2), and author keywords (Supplementary Material 3). Variations referring to the same author, country, or keyword were merged into standardized terms, and the resulting thesaurus files were manually reviewed before analysis to improve consistency and data accuracy.

## Results

**Fig. 2:**
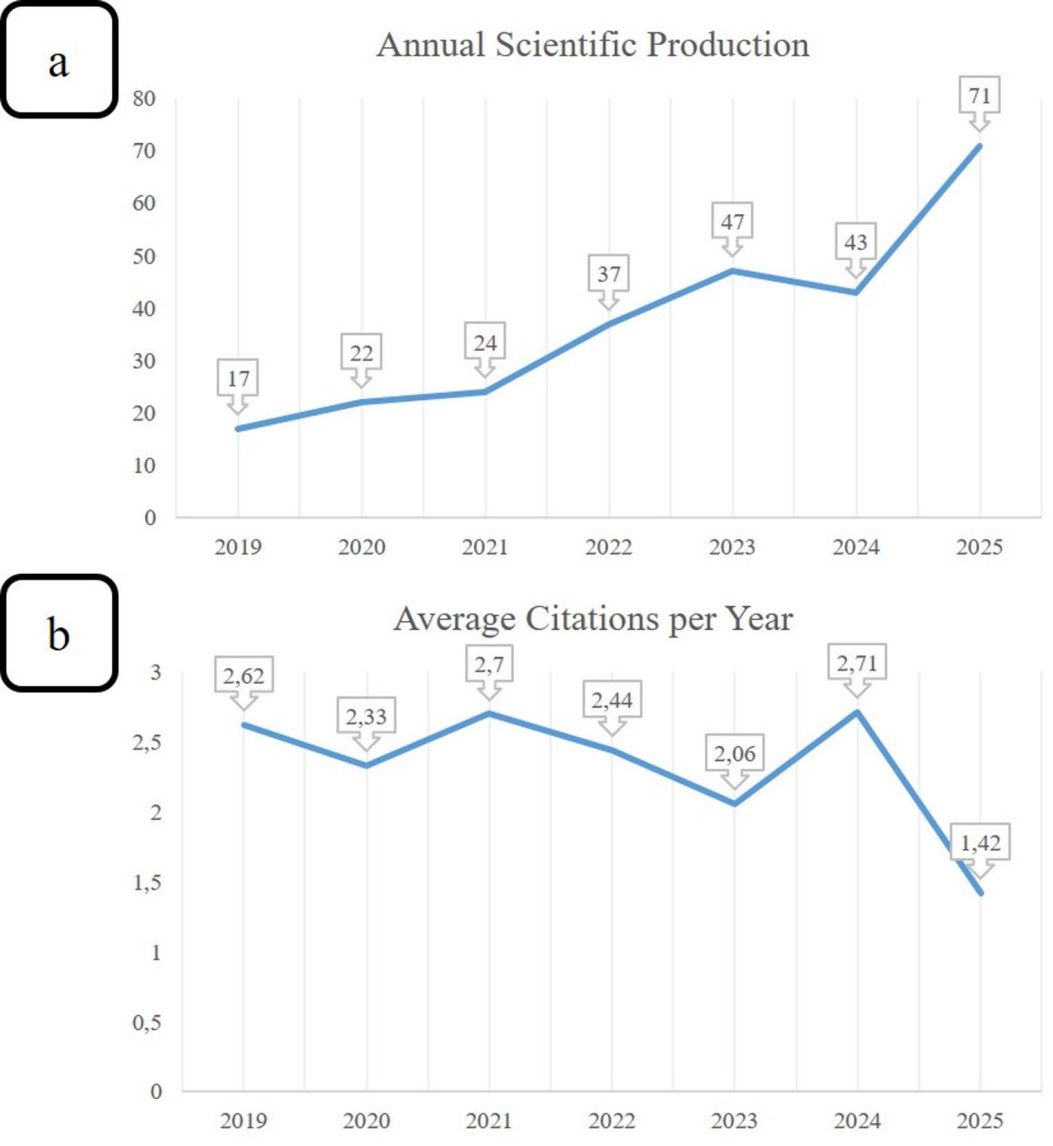
(**a**) Annual scientific production, (**b**) Average citations per year

A total of 261 publications were identified between 2019 and 2025. The annual scientific production demonstrated a clear upward trend over time, increasing from 17 publications in 2019 to a peak of 71 in 2025. After a gradual rise between 2019 and 2021, a more pronounced increase was observed from 2021 onward. Although a slight decrease occurred in 2024 (*n* = 43) compared to 2023 (*n* = 47), this was followed by a substantial rise in 2025 (Fig. 2a).

Average citation rates per year varied across the study period. The highest value was observed in 2024 (2.71), followed by 2021 (2.70), reaching its lowest level in 2025. (1.42) (Fig. 2b).

**Fig. 3:**
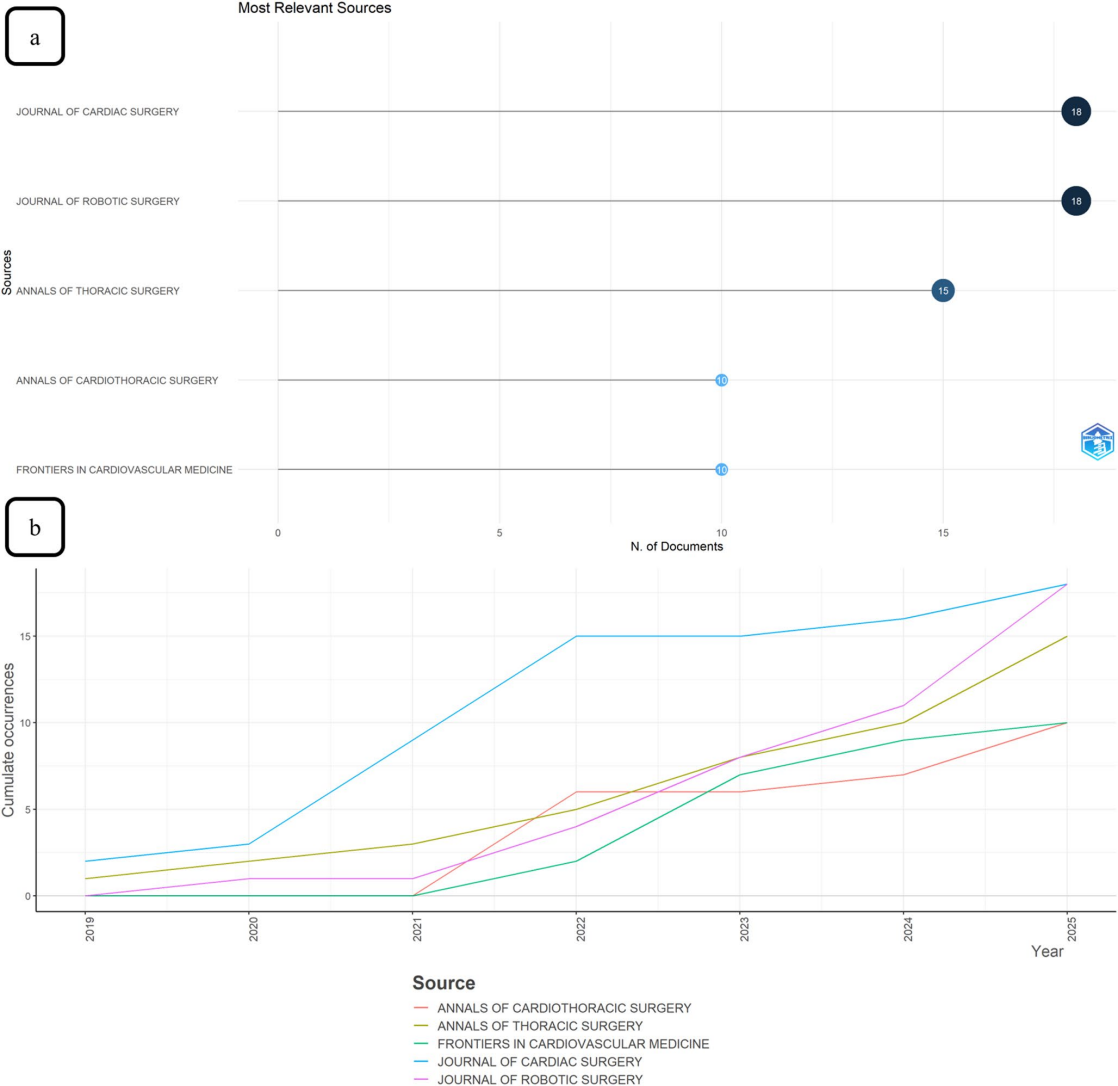
(**a**) Most relevant sources by number of publications, (**b**) Sources’ production over time

A focused assessment of the most relevant sources indicated *Journal of Robotic Surgery* and *Journal of Cardiac Surgery* as the leading journals in the field, each contributing 18 publications (Fig. 3a). Both journals demonstrated a notable increase in productivity over time. *Journal of Robotic Surgery* showed limited activity before 2021, followed by a marked and sustained rise, particularly after 2021, reaching its highest output in 2025. Similarly, *Journal of Cardiac Surgery* exhibited a steady and consistent growth throughout the study period, reflecting its continued contribution to the field (Fig. 3b). *Annals of Thoracic Surgery* followed with 15 publications, while *Annals of Cardiothoracic Surgery* and *Frontiers in Cardiovascular Medicine* each contributed 10 articles.

**Fig. 4:**
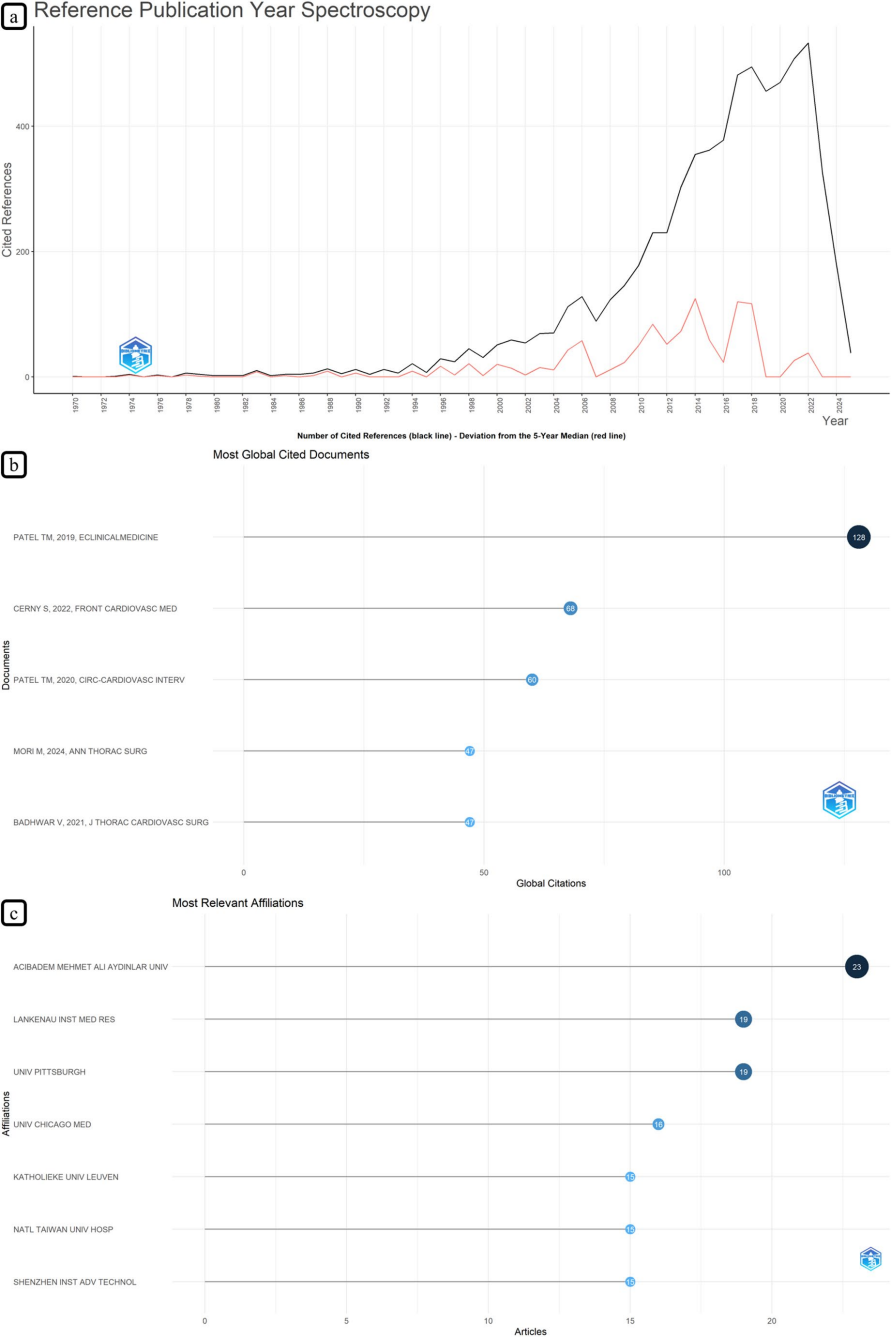
(**a**) Reference publication year spectroscopy, (**b**) Most global cited documents, (c) Most relevant affiliations

RPYS analysis showed that cited references were limited before the 1990s and began to increase gradually after the early 2000s (Fig. 4a). A marked rise was observed after 2010, with the highest number of cited references concentrated between 2015 and 2022. Several peaks were identified in the deviation curve during this period.

The most globally cited document was by *Patel TM (2019)* with 128 citations, followed by *Cerny S (2022)* with 68 citations and *Patel TM (2020)* with 60 citations. *Mori M (2024)* and *Badhwar V (2021)* each received 47 citations (Fig. 4b) [9–13].

In terms of affiliations, *Acibadem Mehmet Ali Aydinlar University* ranked first with 23 publications, followed by *Lankenau Institute for Medical Research* and the *University of Pittsburgh* with 19 publications each. The *University of Chicago Medicine* contributed 16 publications (Fig. 4c).

**Fig. 5:**
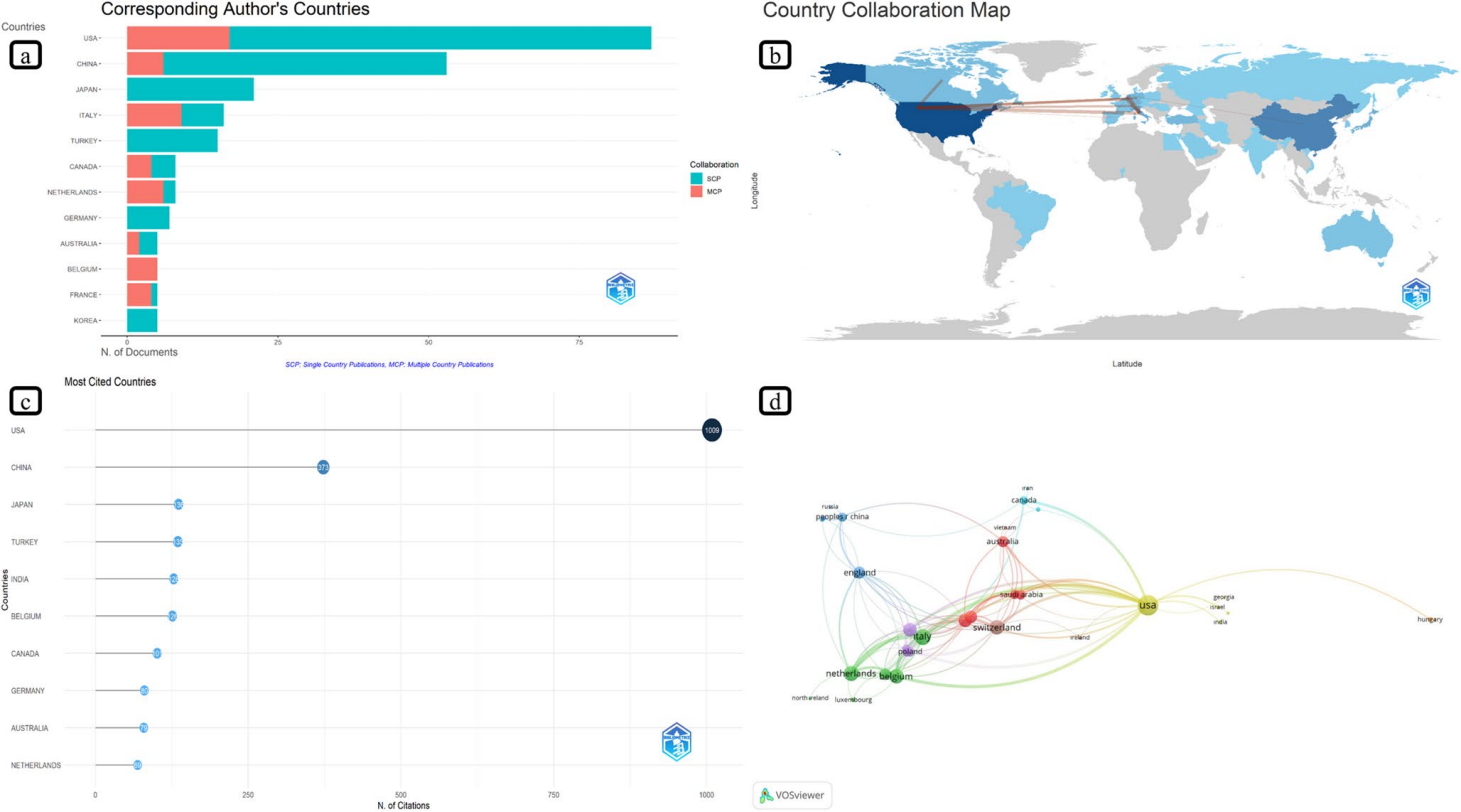
(**a**) Corresponding authors’ countries showing single-country publications (SCP) and multiple-country publications (MCP), (**b**) International country collaboration map, (c) Most cited countries, (d) Country co-authorship network (minimum one document per country). In the network visualization, node size reflects total link strength (TLS), links represent collaboration relationships, and colors indicate different collaboration clusters generated by VOSviewer

The analysis of corresponding author’s countries showed that the United States (USA) ranked first with 87 publications, followed by China (*n* = 53) and Japan (*n* = 21). Italy (*n* = 16) and Turkey (*n* = 15) were also among the leading contributors (Fig. 5a). Across countries, SCP were more prevalent than MCP. USA (SCP = 70, MCP = 17) and China (SCP = 47, MCP = 6) were predominantly driven by SCP, whereas Italy (SCP = 7, MCP = 9) demonstrated a relatively higher proportion of MCP.

USA showed the highest number of collaborations, particularly with Italy (*n* = 7), Canada (*n* = 6), Belgium (*n* = 6), Germany (*n* = 5), Switzerland (*n* = 5), and Spain (*n* = 4). Italy also had strong collaborations with the Netherlands (*n* = 8), Belgium (*n* = 7), and France (*n* = 4) (Fig. 5b).

The most cited countries analysis indicated that USA had the highest citation count (*n* = 1009), followed by China (*n* = 373), while other countries contributed comparatively lower citation counts (Fig. 5c).

The country-level collaboration network is depicted in Fig. 5d. Out of 39 identified countries, 34 showed evidence of collaboration and were subsequently included in the co-authorship analysis. The resulting network comprised 8 clusters and 131 links, with a TLS of 234. In the country-level co-authorship analysis, USA ranked first in total link strength (TLS = 63; *n* = 100 documents), followed by Italy (TLS = 40; *n* = 25) and the Netherlands (TLS = 34; *n* = 16). Switzerland (TLS = 33; *n* = 8) and Belgium (TLS = 32; *n* = 15) were also among the leading countries.

**Fig. 6:**
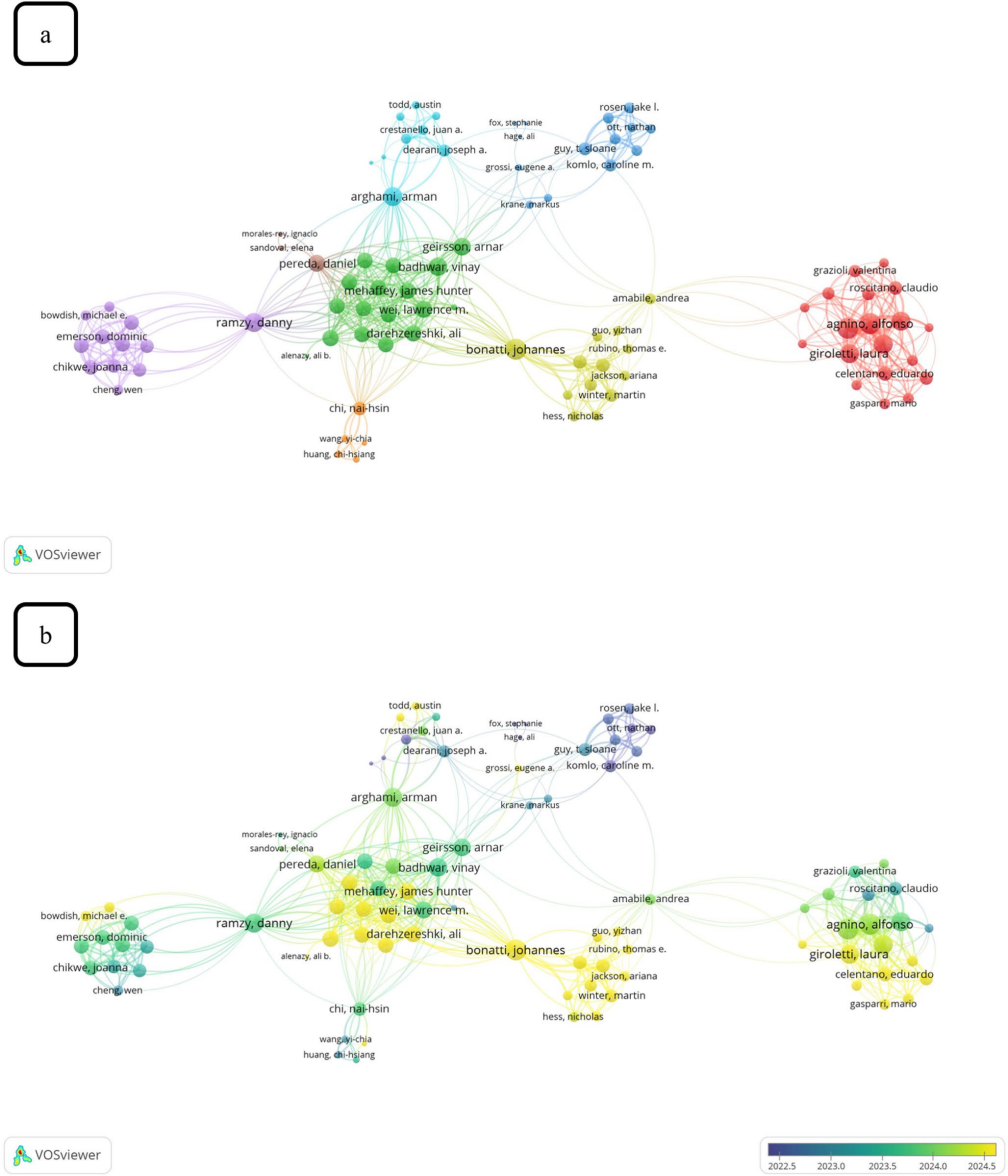
(**a**) Author co-authorship network visualization (minimum two documents per author). Node size reflects total link strength (TLS), links indicate co-authorship relationships, and colors represent collaboration clusters identified by VOSviewer. (**b**) Overlay visualization of the co-authorship network based on average publication year (APY); colors indicate temporal distribution, with blue representing earlier publications and yellow representing more recent publications

The co-authorship network is illustrated in Fig. 6a, and its temporal distribution is presented in Fig. 6b. Overall, 339 authors were identified, of whom 92 met the predefined inclusion criteria and were subsequently incorporated into the co-authorship analysis. The final network comprised 8 clusters and 546 links, with a TLS of 1,143. Sutter FP had the highest TLS (88), followed by Ramlawi B (TLS = 84), Sicouri S (TLS = 84), Werten MC (TLS = 83), and Bonatti J (TLS = 68). Overlay visualization based on APY indicated that the majority of active authors were concentrated in the most recent period, particularly between 2023 and 2025.

**Fig. 7:**
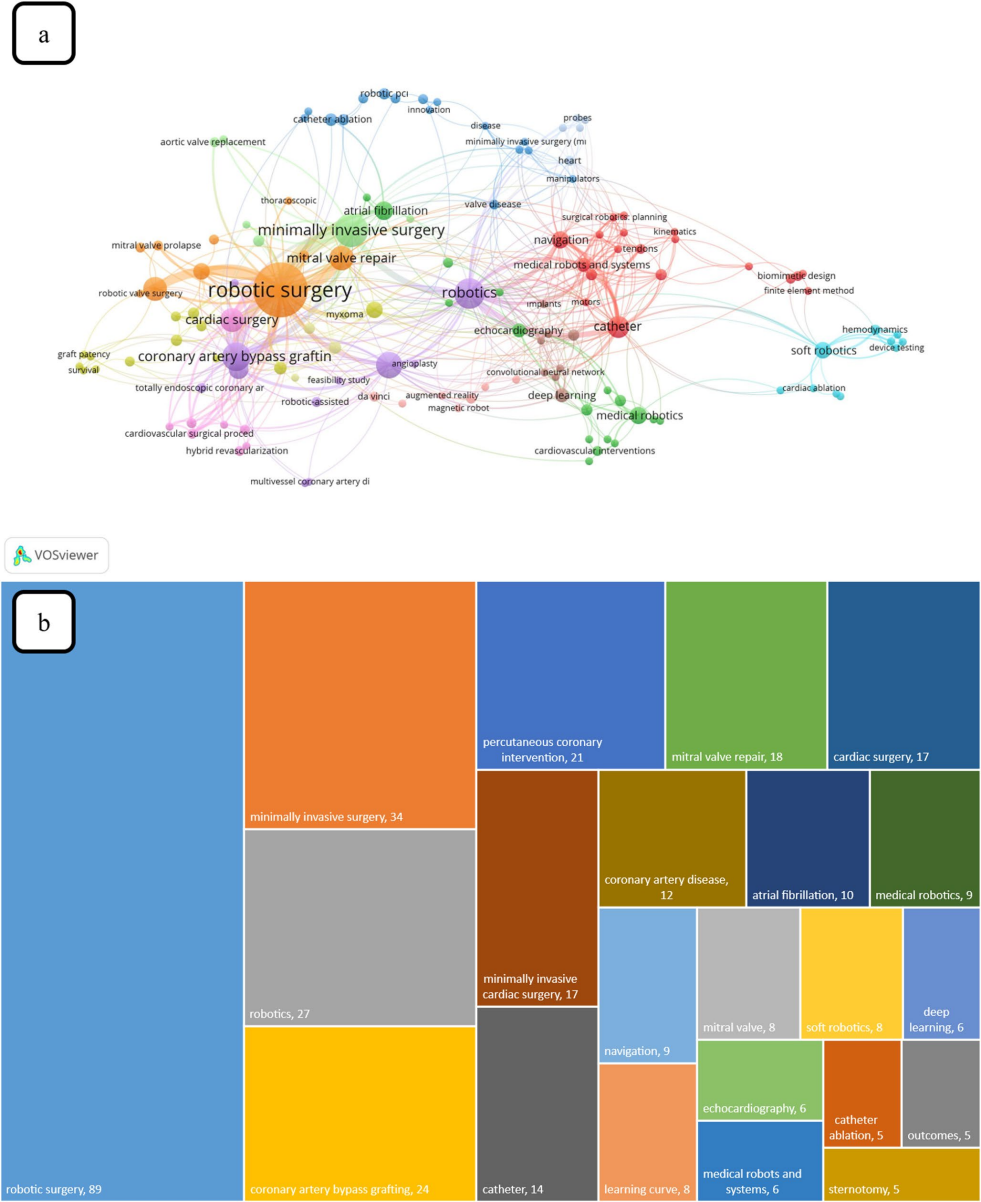
(**a**) Keyword co-occurrence network based on author keywords (minimum two occurrences). Node size reflects keyword occurrence frequency, links indicate co-occurrence relationships, and colors represent keyword clusters identified by VOSviewer. (**b**) Treemap of author keywords with ≥ 5 occurrences

The keyword co-occurrence analysis revealed 135 interrelated keywords grouped into 13 clusters, with a TLS of 902 (Fig. 7a). The most frequently occurring keyword was *robotic surgery* (*n* = 89; TLS = 166), followed by *minimally invasive surgery* (*n* = 34; TLS = 88), *robotics* (*n* = 27; TLS = 101), *coronary artery bypass grafting* (*n* = 24; TLS = 74), and *percutaneous coronary intervention* (*n* = 21; TLS = 48). A treemap visualization was generated for keywords with a minimum of five occurrences, as shown in Fig. 7b.

## Discussion

This bibliometric analysis reveals global research trends in robotic applications in cardiovascular medicine between 2019 and 2025. The marked increase in annual scientific output, particularly after 2021, suggests growing scientific interest and research activity in robotic applications within cardiovascular medicine. However, the fluctuating pattern in average citation counts indicates that the field is still evolving and that the impact of recent studies is likely to increase over time. Keyword analysis demonstrates that research has predominantly focused on minimally invasive surgery, coronary revascularization, and robot-assisted interventions. Analysis of citation patterns indicates that earlier studies tend to have higher average citation counts, implying that they laid the conceptual groundwork of the field. By comparison, reduced citation counts in recent publications are likely a consequence of insufficient time for citation accumulation, commonly referred to as the citation window effect.

Journal analysis identified the *Journal of Robotic Surgery* and the *Journal of Cardiac Surgery* as the most productive sources in this field, both demonstrating a notable increase in publication output in recent years. This pattern highlights that robotic cardiovascular research is concentrated around specific journals and that the field is gradually developing a more structured literature network. Reference year spectrum analysis, showing a clear increase after 2010 and a concentration during the 2015–2022 period, provides additional evidence that robotic cardiovascular applications represent a relatively recent but rapidly expanding area of research. This trend may reflect the growing academic attention devoted to robotic cardiovascular applications in recent years.

Keyword and conceptual structure analyses clearly demonstrate the areas in which robotic applications are concentrated within cardiovascular medicine. The prominence of terms such as “*robotic surgery*,” “*minimally invasive surgery*,” “*coronary artery bypass grafting*,” and “*percutaneous coronary intervention*” indicates that robotic technologies are primarily integrated into revascularization strategies. In addition, the clustering of terms such as “*robotics*,” “*catheter*,” “*medical robotics*,” and “*navigation*” highlights that the field is not limited to surgical applications but is also closely linked to interventional cardiology and device technologies.

The concentration of publications within a limited number of journals and the prominence of specific keyword clusters suggest an increasingly structured research landscape. In particular, the dominance of keywords related to coronary revascularization and minimally invasive surgery indicates that these areas currently represent the principal focus of robotic cardiovascular research. At the same time, the relatively lower representation of structural heart interventions and other emerging applications may highlight potential areas for future investigation and scientific development.

Country-based analyses reveal the dominant roles of USA and China, suggesting that developments in this field are largely driven by these countries. The predominance of single-country publications indicates that research is still largely conducted at the national level, whereas strong collaborations, particularly between USA and European countries, point to an increasingly global research landscape. Similarly, author collaboration analysis demonstrates densely connected networks clustered around specific researchers.

Beyond simple publication volume, the observed geographic concentration of research output suggests that the development of robotic cardiovascular technologies remains closely linked to access to advanced healthcare infrastructure, technological resources, and specialized training programs. The dominant contributions of the United States and China may reflect their substantial investments in medical technology and innovation. Furthermore, the strong international collaboration networks identified among North American and European countries indicate that knowledge generation in this field is increasingly supported by multicenter and cross-border partnerships, which may facilitate the dissemination and standardization of emerging robotic techniques.

These bibliometric findings gain further significance when interpreted alongside the clinical literature. In robotic cardiac surgery, outcomes have been shown to depend largely on institutional experience, with better results reported in high-volume centers [14]. However, the ability of experienced centers to achieve comparable success rates even at lower volumes [15] supports the critical role of technical expertise. Although long-term outcomes of robotic and conventional CABG are similar [16], previous clinical studies have reported potential advantages associated with the less invasive nature of robotic approaches, which may partly explain the prominence of minimally invasive surgery within the literature. This may also be considered to be associated with reduced bleeding and shorter hospital stay.

This trend in coronary revascularization is also consistent with findings showing that robotic CABG is associated with lower target lesion revascularization rates [17] and robotic totally endoscopic coronary artery bypass (TECAB) procedures demonstrate high graft patency [18]. Similarly, hybrid approaches combining robotic surgery and PCI have been associated with high rates of complete revascularization and favorable clinical outcomes [19], which may explain the increasing research interest in these strategies. The lower complication rates reported in female patients further support this trend [20].

The growing research interest in robotic applications in interventional cardiology is consistent with our bibliometric findings. The high success rates of RA-PCI [21, 22] and the reduction in operator radiation exposure [23] help explain the growing role of this technology in clinical practice. Nevertheless, issues related to device compatibility and technical limitations [22, 24] indicate that development in this area is not yet complete. The high success rates observed in PCI highlight the potential for further investigation of robotic applications in other structural and congenital cardiac interventions.

Advances in the field of structural heart disease are also aligned with the bibliometric findings. Parallel to the increase in research activity in this area, the demonstration of comparable early outcomes between robotic and transcatheter valve approaches [25], along with reductions in procedural time achieved through technical improvements [26], suggests an expanding clinical role for robotic technologies. Furthermore, evidence demonstrating that robotic approaches are safe and effective across different cardiac pathologies [27] indicates that these technologies are being evaluated across a broader cardiovascular spectrum, not limited to coronary disease.

Emerging developments such as telerobotic interventions [28] and advances in robotic surgery across various fields [29] represent areas of growing research interest within cardiovascular medicine.

A key strength of the present study is the integration of a large dataset with an extensive analytical approach. Nevertheless, certain limitations should be recognized. The reliance solely on the Web of Science database may have excluded relevant studies indexed in other sources such as Scopus and PubMed, especially in fast-evolving surgical areas. In addition, the restriction to original articles may have resulted in the exclusion of influential reviews, guidelines, consensus statements, and other non-article publications that contribute to the intellectual structure of the field. Therefore, citation patterns and thematic relationships identified in the present study should be interpreted within the context of article-based scientific production. In addition, restricting the dataset to English-language publications may have introduced language bias. Furthermore, despite the use of a comprehensive search strategy, the keyword-dependent nature of literature retrieval may have resulted in the underrepresentation of certain emerging or highly specialized areas of robotic cardiovascular medicine. In addition, citation-based indicators may have been influenced by author self-citations or institutional citation networks, which were not separately evaluated in the present analysis. Citation-based metrics can also be influenced by factors including the timing of publication and the visibility of individual studies and should thus be interpreted with caution. Additionally, citation analyses may be affected by citation window bias, as more recent publications, particularly those from 2025, have had limited time to accumulate citations. Although average citations per year were used to partially address this issue, citation-based comparisons should be interpreted with caution.

In conclusion, robotic applications in cardiovascular medicine represent an increasingly active area of research characterized by growing scientific output and expanding international collaboration. While robotic systems offer significant clinical advantages, further studies are needed to overcome existing technical and economic limitations. Future research should also prioritize international collaboration, procedural standardization, and the evaluation of emerging robotic applications within cardiovascular medicine.

## Supplementary Information

Below is the link to the electronic supplementary material.

Supplementary Material 1

Supplementary Material 2

Supplementary Material 3

Supplementary Material 4

## Data Availability

The detailed dataset is provided as Supplementary Data 4.
